# Interactions between SARS-CoV-2 and influenza, and the impact of coinfection on disease severity: a test-negative design

**DOI:** 10.1093/ije/dyab081

**Published:** 2021-05-03

**Authors:** Julia Stowe, Elise Tessier, H Zhao, Rebecca Guy, Berit Muller-Pebody, Maria Zambon, Nick Andrews, Mary Ramsay, Jamie Lopez Bernal

**Affiliations:** Immunisation and Countermeasures Division, National Infection Service, Public Health England, London, UK; Immunisation and Countermeasures Division, National Infection Service, Public Health England, London, UK; Immunisation and Countermeasures Division, National Infection Service, Public Health England, London, UK; Healthcare Associated Infections and Antimicrobial Resistance Division, National Infection Service, Public Health England, London, UK; Healthcare Associated Infections and Antimicrobial Resistance Division, National Infection Service, Public Health England, London, UK; Virus Reference Department, National Infection Service, Public Health England, London, UK; Statistics, Modelling and Economics Department, National Infection Service, Public Health England, London, UK; Immunisation and Countermeasures Division, National Infection Service, Public Health England, London, UK; Immunisation and Countermeasures Division, National Infection Service, Public Health England, London, UK

**Keywords:** SARS-CoV-2, influenza, coinfection, interaction, epidemiology

## Abstract

**Background:**

The impact of SARS-CoV-2 alongside influenza is a major concern in the northern hemisphere as winter approaches.

**Methods:**

Test data for influenza and SARS-CoV-2 from national surveillance systems between 20 January 2020 and 25 April 2020 were used to estimate influenza infection on the risk of SARS-CoV-2 infection. A test-negative design was used to assess the odds of SARS-CoV-2 in those who tested positive for influenza compared with those who tested negative. The severity of SARS-CoV-2 was also assessed using univariable and multivariable analyses.

**Results:**

The risk of testing positive for SARS-CoV-2 was 58% lower among influenza-positive cases and patients with a coinfection had a risk of death of 5.92 (95% confidence interval: 3.21–10.91) times greater than among those with neither influenza nor SARS-CoV-2. The odds of ventilator use or death and intensive care unit admission or death were greatest among coinfected patients.

**Conclusions:**

Coinfection of these viruses could have a significant impact on morbidity, mortality and health-service demand.

## Background

It is likely that both SARS-CoV-2 and seasonal respiratory pathogens, most notably influenza, will be co-circulating as the northern-hemisphere 2020/2021 winter approaches. The potential impact of COVID-19 alongside influenza on morbidity, mortality and health-service capacity is a major concern, although, currently, little is understood about the interaction between these two respiratory viruses.^1^^,^^2^

There is existing evidence of pathogenic competition between respiratory viruses, including between influenza and seasonal coronaviruses.^3^^,^^4^ This could be through immune-mediated interference resulting in some viruses diminishing during the peak of another virus—a phenomenon that has been recognized for many decades.^3^^,^^5^^,^^6^ To date, there is some evidence of ectopic interaction between the SARS-CoV-2 protein and host proteins,^7^ although there is no information on the pathogenic interaction between SARS-CoV-2 and influenza, and the epidemiological impact of such an interaction is unknown.

**Key Messages**

 

If individuals are coinfected with both SARS-CoV-2 and influenza, this could lead to more severe disease outcomes. Since the beginning of the 2020 SARS-CoV-2 pandemic, a number of case reports of SARS-CoV-2 and influenza coinfection with severe outcomes have been published.^1^^,^^8–12^ However, there is a propensity for case reports to highlight more severe cases and there has been no systematic analysis of disease outcomes in coinfected patients compared with those in non-coinfected controls.

- The potential impact of COVID-19 alongside influenza on morbidity, mortality and health-service capacity is a major concern and little is understood about the interaction between these two respiratory viruses.
- We evaluated the interaction between influenza and SARS-CoV-2 in England. We found that SARS-CoV-2 was 58% lower among influenza cases. Coinfection patients had a 5.92 (95% confidence interval: 3.21–10.91) greater risk of death than patients with neither influenza nor SARS-CoV-2.
- As the 2020–2021 northern-hemisphere influenza season approaches, testing strategies should include testing for both influenza and SARS-CoV-2, and maximizing the uptake of influenza vaccination, particularly in groups at higher risk of both diseases.

In the UK, the 2019–2020 influenza season peaked early, with activity declining significantly from January 2020.^13^ The season saw lower activity with influenza A(H3N2) as the predominant strain.^13^ The first SARS-CoV-2 infection occurred in late January 2020 arising from an imported case and the distribution of SARS-CoV-2 rose with sustained community transmission from early March in the UK, peaking on 7 April 2020 with 4493 cases and, on 21 April, the total number of daily SARS-CoV-2 deaths peaked at 1172.^14^ As such, there was only a limited period of overlap between influenza circulation and SARS-CoV-2 circulation. In this study, we explore the interaction between influenza and SARS-CoV-2 during the latter stages of the 2019–2020 influenza season in England.

The aims of the study are two-fold: first, to assess whether infection with influenza is associated with a reduced risk of SARS-CoV-2 infection and, second, to assess whether coinfection with influenza is associated with a more severe SARS-CoV-2 outcome such as death, being admitted to hospital, admitted to an intensive care unit (ICU) or requiring ventilatory support.

## Methods

### Data sources and data linkage

The SGSS (Second Generation Surveillance System) and the Respiratory DataMart System (RDMS) were used to obtain all influenza-positive cases between 01 January 2020 and 2 June 2020.^15^^,^^16^ In England, diagnostic testing for influenza is done almost exclusively by PCR. For the analyses, data were restricted to the time period between 20 January 2020 up to 25 April 2020, when the first SARS-CoV-2 and influenza coinfection occurred and the last influenza sample was reported in the RDMS. Individuals tested for influenza who had a negative result in the RDMS were also extracted. Both groups were matched to SARS-CoV-2 test results (positive and negative) in SGSS as of 2 June 2020. Cases were matched using a valid patient NHS (National Health Service) number (unique patient identifier in England; complete in 91.5% of test results) and, where not available, patient’s date of birth, forename and surname (complete for 95.1% of cases in which the NHS number was not available). All individuals who were sampled within 7 days of each other for SARS-CoV-2 and influenza test were included in the study. A coinfection was defined as testing positive for both influenza and SARS-CoV-2 within 7 days of each other’s sample date.

All individuals tested for influenza and SARS-CoV-2 (positive and negative results) were linked to the Demographic Batch Service (DBS)—a national database coordinated by NHS digital that allows the tracing of information against personal demographics—and the date of death was extracted.^17^ Additionally, individuals who tested positive for SARS-CoV-2 were matched to the Public Health England COVID-19 death data set that has been updated daily throughout the pandemic. Deaths from 6 days before to 28 days after the test result were included.

Test results were also linked to the Secondary Uses Service (SUS) data set and the Hospital Episode Statistics (HES) data set, which contain information on all admitted patient care, outpatient and accident-and-emergency-service attendances at NHS hospitals in England.^18^^,^^19^ These data sets were used to identify patients in an ICU and who required the use of a ventilator within 14 days before to 28 days after the earliest test-sample date.^20^ The SUS and HES data sets were also used to extract ethnicity and co-morbidity information. Co-morbidities were identified using the International Classification of Diseases 10^th^ revision (ICD-10) codes and grouped into the following categories: asplenia or dysfunction of the spleen, asthma, chronic heart disease, chronic kidney disease, chronic liver disease, chronic neurological disorders, chronic respiratory disease (excluding asthma), dementia including Alzheimer's, diabetes, malignancies affecting the immune system, obesity, other neoplasms, rheumatological diseases, and transplantations and conditions affecting the immune system. For the co-morbidities linkage, data were restricted to inpatient and outpatient hospital episodes in the last 5 years.

### Statistical analysis

#### Effect of influenza infection on the risk of SARS-CoV-2 infection

The total number of positive and negative SARS-CoV-2 and influenza test results from weeks 1 to 17 in 2020 were assessed. Percent positivity was calculated for individuals with a SARS-CoV-2 and influenza coinfection and individuals with no influenza infection by dividing the number of individuals with SARS-CoV-2-positive results by the total number of individuals tested and multiplied by 100. Additionally, the total number of individuals with a SARS-CoV-2 and influenza coinfection were assessed by influenza type.

To estimate the effect of recent influenza infection on the risk of SARS-CoV-2 infection, a test-negative design was used. Univariable and multivariable analyses on the odds of SARS-CoV-2 in those who tested positive for influenza compared with those who tested negative for influenza were conducted adjusting for age, sex, ethnicity, region, co-morbidity and sample week. The model was rerun for individuals who were sampled on the same day for influenza and SARS-CoV-2. Finally, to determine the influence of unmeasured confounding such as occupation, the analysis was stratified by age into children (<19 years old), working-age adults (19–65 years old) and older adults (>65 years old).

#### Severity and risk of death among individuals with a coinfection

The mortality rate among individuals with a SARS-CoV-2 and influenza coinfection and those with SARS-CoV-2 infection who tested negative for influenza was calculated by dividing the number of deaths by the total number of individuals tested by age group.

To assess whether having a coinfection was associated with death, univariable and multivariable analyses on the odds of death adjusted for age, sex, ethnicity, co-morbidity (0 or 1+) and coinfection status (influenza-negative/SARS-CoV-2-negative; influenza-negative/SARS-CoV-2-positive; influenza-positive/SARS-CoV-2-negative; influenza-positive/SARS-CoV-2-positive) was assessed. This analysis was repeated with a composite outcome of ventilator use or death use and a composite outcome of ICU admission or death.

## Results

**Figure 1:**
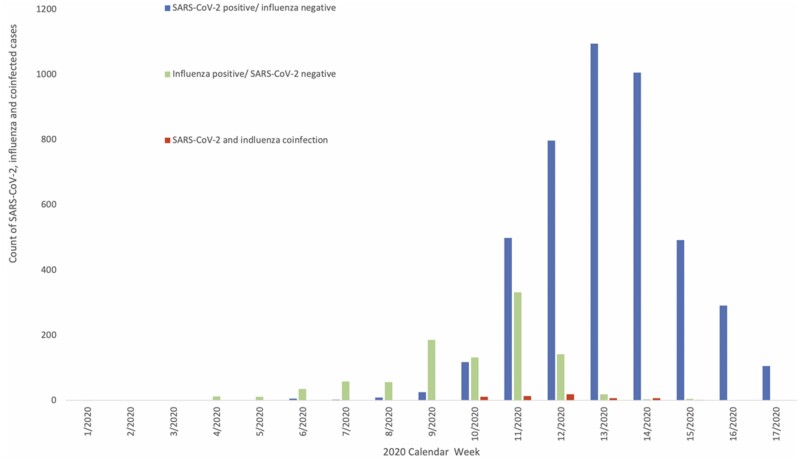
Distribution of SARS-CoV-2, influenza and coinfected cases in England from between 20 January 2020 and 25 April 2020 (Weeks 1–17)

A total of 19 256 individuals were tested for both influenza and SARS-CoV-2 between 20 January 2020 and 25 April 2020, when the last positive influenza test was detected in the RDMS. Of these individuals, 16 764 (87.1%) sampled for both influenza and SARS-CoV-2 on the same day and the remaining 2492 (12.9%) were sampled within 7 days of each other. In total, 58 individuals had a SARS-CoV-2 and influenza coinfection, 992 had a positive influenza result and were negative for SARS-CoV-2, 4443 had a positive SARS-CoV-2 result and were negative for influenza, and the remaining 13 763 were negative for both SARS-CoV-2 and influenza during this period (Figure 1). Of the 58 patients with a SARS-CoV-2 and influenza coinfection, 32 (55.2%) cases were aged ≥70 years. Of the 58 cases with a SARS-CoV-2 and influenza coinfection, 31 individuals had influenza A (not subtyped), 8 had influenza A(H1N1), 16 had influenza B, 1 had influenza A&B and 2 cases had unknown influenza type. All individuals were tested for influenza by PCR. Week 12 had the highest reported SARS-CoV-2 and influenza coinfections (20 individuals, Table 1).

**Table 1:** SARS-CoV-2 positivity among influenza cases and influenza-test negatives by sample week in England from 20 January 2020 to 25 April 2020

Week	Influenza-positive				Influenza-negative			
	SARS-CoV- 2-negative	SARS-CoV- 2-positive	Total	SARS-CoV-2 positivity (%)	SARS-CoV- 2-negative	SARS-CoV- 2-positive	Total	SARS-CoV-2 positivity (%)
1	1	0	1	0	0	0	0	–
2	0	0	0	–	1	0	1	0
3	0	0	0	–	4	0	4	0
4	12	0	12	0	34	0	34	0
5	11	0	11	0	60	0	60	0
6	34	0	34	0	323	6	329	1.8
7	58	0	58	0	583	2	585	0.3
8	53	0	53	0	329	9	338	2.7
9	189	0	189	0	1471	22	1493	1.5
10	125	11	136	8.1	1211	113	1324	8.5
11	330	12	342	3.5	2957	483	3440	14.0
12	145	20	165	12.1	2322	783	3105	25.2
13	25	6	31	19.4	1342	1092	2434	44.9
14	4	8	12	66.7	1072	1022	2094	48.8
15	4	1	5	20.0	839	496	1335	37.2
16	0	0	0	–	758	303	1061	28.6
17	1	0	1	0	451	111	562	19.8

When observing descriptive characteristics by the four test-result outcomes, 31.0% of the coinfected were aged ≥80 years, which was similar to those testing positive for SARS-CoV-2 and negative for influenza (29.8%) but greater than those testing negative for both (15.4%) or for those testing positive for influenza but negative for SARS-CoV-2 (11.9%) (Supplementary Table S1, available as Supplementary data at *IJE* online). There was a similar distribution of male and females for all groups and the dominant ethnicity was White for all test-result categories, followed by Asian/Asian British apart for those with a coinfection, for which the second most common ethnicity was Black/Black British (Supplementary Table S1, available as Supplementary data at *IJE* online).

A total of 13 451 (69.9%) individuals linked to a hospital-admission record in SUS between 1 December 2020 and 24 August 2020 of whom 12 253 individuals had an associated record in the 14 days before and ≤28 days after the earliest SARS-CoV-2 or influenza test date. Of the 12 253 individuals, a total of 1666 (13.6%) individuals had an ICU admission and 890 (7.3%) were ventilated (Supplementary Figure S1, available as Supplementary data at *IJE* online). Of the 19 256 individuals, 2469 (12.8%) died, of whom 25/58 (43.1%) of the SARS-CoV-2 and influenza coinfected cases died.

### Effect of influenza infection on the risk of SARS-CoV-2 infection

SARS-CoV-2 positivity among influenza-positive cases was generally lower than SARS-CoV-2 positivity among influenza-test negatives (Table 1). The highest SARS-CoV-2-positivity rate for both influenza-positive and -negative cases was in Week 14 (66.7% and 44.8%, respectively).

After adjusting for age, sex, ethnicity, region, co-morbidity and sample week in the multivariable analysis, the results indicate that the odds of testing positive for SARS-CoV-2 were 58% lower among influenza-positive cases [odds ratio (OR) 0.42, 95% confidence interval (CI): 0.31–0.56] (Table 2). Using only those testing for SARS-CoV-2 and influenza (*n* = 16 764) resulted in an odds ratio of 0.44 (95% CI: 0.31–0.61, *p* < 0.001). After stratifying by age into children (under 19 years old), working-age adults (19–65 years old) and older adults (>65 years old), the working-age and older population had lower odds of SARS-CoV-2 if testing positive for influenza (OR 0.26, 95% CI: 0.15–0.45, *p* < 0.001) and (OR 0.52, 95% CI: 0.35–0.75, *p* = 0.001), respectively. Conversely, there was no association between influenza positivity and SARS-CoV-2 positivity among children (OR 1.07, 95% CI: 0.38–3.01, *p* = 0.897), although the numbers were small in this age group. To formally test the interaction between influenza and the stratified age cohorts, the model was fitted with separate terms for the age cohorts resulting in no evidence of interaction (*p* = 0.01).

**Table 2:** Odds of SARS-CoV-2 infection by influenza status stratified by age (England from 20 January 2020 to 25 April 2020)^a^

Age group	Count coinfection	Characteristic		Unadjusted odds ratio	95% CI	Adjusted odds ratio	95% CI
Overall	58	Influenza status	Negative	Baseline		Baseline	
			Positive	0.18	(0.14–0.24)	0.42	(0.31–0.56)
Under 19 years old	5	Influenza status	Negative	Baseline		Baseline	
			Positive	0.55	(0.22–1.38)	1.07	(0.38–3.01)
Working-age	17	Influenza status	Negative	Baseline		Baseline	
			Positive	0.12	(0.07–0.20)	0.26	(0.15–0.45)
Older population	36	Influenza status	Negative	Baseline		Baseline	
			Positive	0.29	(0.20–0.41)	0.52	(0.35–0.75)

a Adjusted for age, sex, co-morbidity, region, ethnicity and sample week.

### Risk of death among individuals with a coinfection

After linking all individuals to the death data sets, a total of 2699 individuals had a recorded death with a SARS-CoV-2 or influenza test (positive or negative) within 28 days before and 6 days after the death date. Of the reported deaths, 25 (0.9%) individuals had a SARS-CoV-2 and influenza coinfection, 1419 (52.6%) had a SARS-CoV-2 infection only, 48 (1.8%) had influenza only and 1206 (44.7%) had neither SARS-CoV-2- nor influenza-positive results.

Overall, 43.1% of the cases with coinfection died compared with 26.9% of those who tested positive only for SARS-CoV-2 (Table 3). Age-specific mortality rates were higher among older people with a SARS-CoV-2 and influenza coinfection (Table 3). For individuals with influenza only, the overall mortality rate was 48/992 = 4.8%, and for those negative for both, the mortality rate was 1203/13 763 = 8.7%.

**Table 3:** SARS-CoV-2 and influenza coinfection deaths and mortality rate (%) and SARS-CoV-2 with no influenza deaths and mortality rate (%) by age groups in England from 20 January 2020 to 25 April 2020

Age (years)	Coinfection (flu-positive and SARS-CoV-2-positive) *n* = 58					Single infection (SARS-CoV-2-positive and flu-negative)				
	Total	Died	ICU admission	Mortality rate (%)	ICU rate (%)	Total	Died	ICU admission	Mortality rate (%)	ICU rate (%)
<5	0	0	0	–	–	37	0	2	0.0	5.4
5–9	2	0	0	0.0	0.0	7	0	0	0.0	0.0
10–19	3	0	0	0.0	0.0	33	1	4	3.0	12.1
20–29	1	0	0	0.0	0.0	162	4	10	2.5	6.2
30–39	7	1	2	14.3	28.6	295	5	33	1.7	11.2
40–49	2	0	0	0.0	0.0	426	21	80	4.9	18.8
50–59	5	1	3	20.0	60.0	658	81	158	12.3	24.0
60–69	6	3	1	50.0	16.7	670	155	160	23.1	23.9
70–79	14	8	1	57.1	7.1	824	307	117	37.3	14.2
80+	18	12	0	66.7	0.0	1331	619	15	46.5	1.1
Total	58	25	7	43.1	12.1	4443	1193	581	26.9	13.1

**Figure 2:**
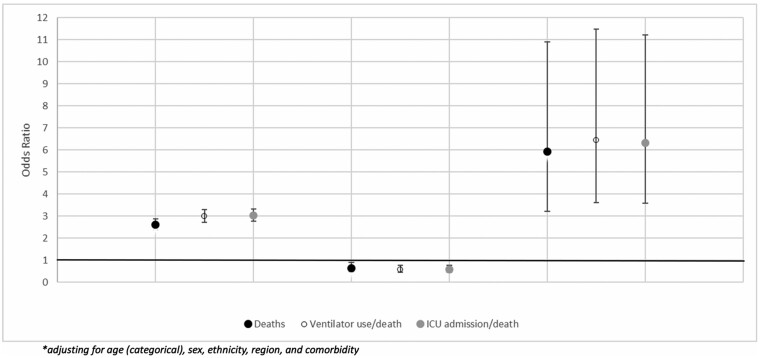
The odds and 95% confidence intervals* of death, ventilator use and ICU admission by influenza/SARS-CoV-2 status compared with individuals with no infection in England from 20 January 2020 to 25 April 2020

The multivariable analysis adjusting for age, sex, ethnicity, co-morbidity (0 or 1+) and coinfection status (flu-negative/SARS-CoV-2-negative; flu-negative/SARS-CoV-2-positive; flu-positive/SARS-CoV-2-negative; flu-positive/SARS-CoV-2-positive) indicated that the odds of death were 5.92 (95% CI: 3.21–10.91) times greater among individuals with a SARS-CoV-2 and influenza coinfection than among those with neither influenza nor SARS-CoV-2 and was higher than among those with only SARS-CoV-2 where the odds of death were 2.61 (95% CI: 2.36–2.88) times greater compared with those with no SARS-CoV-2 or influenza. Patients with a SARS-CoV-2 and influenza coinfection were around twice as likely to die (OR 2.27, 95% CI: 1.23–4.19) compared with those with SARS-CoV-2 alone. For those only positive for influenza, there was a slightly decreased mortality risk (OR 0.64, 95% CI: 0.47–0.89) (Figure 2) compared with individuals with no infections. To formally test the interaction between influenza and SARS-CoV-2, the same model was fitted but with separate terms for influenza, SARS-CoV-2 and the interaction of influenza and SARS-CoV-2, which gave an interaction effect (*P* ≤ 0.001) of an additional 3.60 odds of death (95% CI: 1.83–7.11) compared with that expected if influenza and SARS-CoV-2 acted independently.

When combining ventilator use or death into a composite variable, the odds were 6.43 times greater among individuals with coinfection (95% CI: 3.61–11.47). The ICU admission or death composite had odds that were 6.33 times greater among individuals with coinfection (95% CI: 3.57–11.23) (Figure 2). The patients with a SARS-CoV-2 and influenza coinfection were around twice as likely to be ventilated during admission (OR 2.15, 95% CI: 1.20–3.84) or to be admitted to an ICU (OR 2.08, 95% CI: 1.17–3.70) compared with those with SARS-CoV-2 alone. A test for interaction for both the ventilator composite and the ICU composite gave an effect (*P* ≤ 0.001) with an additional 3.38 odds of coinfection (95% CI: 1.81–6.34) and 3.39 odds of coinfection (95% CI: 1.83–6.29), respectively, compared with that expected if influenza and SARS-CoV-2 acted independently.

## Conclusions

We found that influenza infection was associated with a lower risk of SARS-CoV-2 infection, suggesting that there may be pathogenic competition between these two viruses. We also found strong evidence that coinfection with influenza and SARS-CoV-2 was associated with an increased risk of death (OR 5.92, 95% CI: 3.21–10.91) and severe disease, and that this appears to be beyond the additive effect of the two viruses acting independently.

The risk of testing positive for SARS-CoV-2 was 58% lower among influenza-positive cases. This is consistent with recent descriptive evidence from New York where <3% of those testing positive for SARS-CoV-2 had coinfection with influenza whereas 13% of those testing negative for SARS-CoV-2 were influenza-positive.^21^ It is also consistent with existing evidence on the interaction between influenza and seasonal coronavirus and rhinovirus.^3^^,^^4^^,^^22^ There are biologically plausible mechanisms for such an effect, including stimulation of non-specific immune responses by the first infectious agent, such as the induction of a refractory state in bystander cells as a result of the antiviral effect of interferon induced as part of an innate immune response to an RNA viral infection.

Our findings cannot distinguish between a reduced risk of SARS-CoV-2 among those first infected with influenza or vice versa. A recent study has suggested that SARS-CoV-2 has a lower growth rate than influenza and is suppressed if the infections start simultaneously; however, if an influenza infection were to occur after SARS-CoV-2 infection, a coinfection would be detected.^23^ Our findings would not support the relaxation of preventative measures against influenza, including vaccination, given the risk of morbidity and mortality from influenza^24^^,^^25^ as well as our finding of adverse outcomes associated with influenza and SARS-CoV-2 coinfection. Furthermore, results from Brazil indicated a significantly lower odds of needing intensive care treatment, invasive respiratory support and death among patients with SARS-CoV-2 who received the inactivated trivalent influenza vaccine.^26^ The International Council on Adult Immunization highlights in their roadmap that influenza, pneumococcal and herpes zoster vaccine programmes are more urgent than ever before.^27^ As a further potential implication on influenza vaccination, if there is a competitive effect between influenza and SARS-CoV-2, this effect may also be seen with live attenuated influenza vaccination, which, if offered to children in England, could in turn have a role in outbreak management. It is possible that the attenuated influenza virus in the vaccine stimulates a short-term non-specific immune response that provides a temporary protective effect against SARS-CoV-2. Further research on the pathology of influenza and SARS-CoV-2 coinfection such as the order of infection and the effect of timing of influenza infection on the risk of acquiring SARS-CoV-2 infection, as well as any effect of live attenuated influenza vaccination, is required.

The results from this study indicate that the risk of death was nearly six times greater among individuals with a SARS-CoV-2 and influenza coinfection than among those with neither influenza nor SARS-CoV-2 and that this effect is higher than the risk associated with SARS-CoV-2 infection alone. Similarly, the combined outcomes of ventilator use or death and ICU admission or death gave similar results. These findings suggest a possible synergistic effect between SARS-CoV-2 and influenza once an individual is coinfected. The high mortality rate is consistent with case reports of severe outcomes in coinfected patients.^11^^,^^12^^,^^28^ Conversely, some case series have not seen increased severity with influenza and SARS-CoV-2 coinfection, where the outcomes have been similar to cases with SARS-CoV-2 only.^29^^,^^30^ Synergistic effects have previously been reported between influenza and other respiratory viruses, e.g. by facilitating cell-to-cell spread,^31^ as well as in a recent animal study that found increased disease severity among SARS-CoV-2 and pandemic influenza A(H1N1) hamsters compared with those with SARS-CoV-2 infection alone.^32^

Our results found no increased risk associated with ICU admission, ventilator use and death among flu-positive cases. It is likely that this cohort was generally unable to warrant testing for both influenza and SARS-CoV-2 at this early stage of the pandemic before mass testing had become established. The slightly increased odds of poor outcome in those testing negative compared with those positive for influenza alone are likely to be due to the high morbidity even in the baseline group. In this analysis, it is assumed that, within those with respiratory conditions who are being tested for SARS-CoV-2 and influenza (mainly at hospital), the likelihood of testing itself due to the respiratory condition is not dependent on whether the individual has influenza or some other non-COVID condition. It is possible that a negative association between SARS-CoV-2 and influenza could be induced through collider bias if admission (and testing) due to flu is higher than admission and testing due to another condition that is neither influenza nor SARS-CoV-2.

Our findings emphasize the importance of influenza vaccination in at-risk groups and early administration of antivirals where coinfection is identified or suspected. This also adds further weight to the need for effective vaccines against influenza, in particular among the elderly, among whom vaccine effectiveness tends to be lower and among whom most coinfections were seen. This has been an area of development in recent years with the introduction of high-dose and adjuvant vaccines.^33^

Studies of other respiratory viral infections have not indicated adverse outcomes from coinfection; e.g. a study assessing SARS-associated coronavirus and metapneumovirus in Hong Kong showed that there was no significant difference in the outcomes, including deaths between those with a SARS-associated coronavirus and metapneumovirus coinfection vs SARS-associated coronavirus alone.^34^ It is important to note that these are case studies of hospitalized individuals and the comparisons do not adjust for potential confounders.

To our knowledge, our study is the first epidemiological study to use national-level data on both positive and negative SARS-CoV-2 and influenza cases. By extracting all cases with a SARS-CoV-2 and influenza test result and linking the data to HES, we were able to assess the effects of SARS-CoV-2 and influenza coinfections compared with single infection and negative test results while controlling for variables such as ethnicity, co-morbidities, sex and age, which are known factors for SARS-CoV-2 morbidity.^35–37^ Furthermore, the test-negative design controls for the propensity for more severe cases to be tested for other respiratory viruses.

Most of the SARS-CoV-2 tests were collected when the government policy was to test individuals on admission to hospital with lower respiratory tract infections and healthcare workers.^38^ Therefore, the majority of SARS-CoV-2 cases were individuals with moderate to severe symptoms and mild cases are likely to have been missed. Additionally, influenza test results collected from the RDMS are only collected from sentinel laboratories. However, the test-negative controlled design means that none of the study arms was biased towards more severe outcomes, as all were tested for both diseases.

Additionally, in our study, the majority of cases with SARS-CoV-2 coinfection had influenza subtype A. Due to small numbers, it was not possible to determine whether the risk of SARS-CoV-2 coinfection and severity of disease varied by influenza subtype. Whereas, in the 2019–2020 influenza season, the majority of the subtype of the influenza A cases were influenza A(H3N2), towards the end of the season, there was a shift towards influenza A(H1N1), which is consistent with our finding of more influenza A(H1N1) cases among those coinfections that were subtyped.^13^ The impact severity of influenza and SARS-CoV-2 coinfection by different subtypes should be further considered in the upcoming influenza season. Furthermore, the influenza-vaccination status of the patients was not available so we could not adjust for the vaccination status of the patients in the model. Whereas our findings provide evidence of pathogenic competition between influenza and SARS-CoV-2, a significant number of coinfections occur and they appear to be associated with higher mortality rates. Further investigation is needed in order to understand the potential mechanisms for any synergistic interaction.

Co-circulation of these two viruses could have a significant impact on morbidity, mortality and health-service demand. As the 2020–2021 northern-hemisphere influenza season approaches, it is important that a high index of suspicion for coinfection is maintained. Testing strategies should include influenza and other respiratory viruses as well as SARS-CoV-2 and measures should be adopted to prevent coinfection, including maximizing uptake of the influenza vaccination, particularly in groups at higher risk of both diseases.

## Supplementary data

Supplementary data are available at *IJE* online.

## Ethics approval

Public Health England is able to process identifiable data under Regulation 3 of the Health Service (Control of Patient Information) (Secretary of State for Health, 2002). This is for purposes related to communicable diseases and other risks to public health.

## Funding

The work was supported by authors at Public Health England as part of the routine functions of surveillance and control of communicable diseases. Public Health England, National Infection Service, Immunisation and Countermeasures Division has provided vaccine manufacturers with post-marketing surveillance reports, which the Marketing Authorisation Holders are required to submit to the UK licensing authority in compliance with their Risk Management Strategy. A cost recovery charge is made for these reports.

## Data availability

The data underlying this article cannot be shared publicly due to patient confidentiality and deductive disclosure issues. The data will be shared on reasonable request to the corresponding author.

## Author contributions

J.L.B., E.T., J.S. and N.A. developed the study protocol. H.Z., R.G., J.S., E.T. and B.M.P. extracted the data and J.S., E.T., J.L.B. and N.A. conducted the statistical analysis. The following authors assisted in the interpretation of the data: E.T., J.S., N.A., J.L.B., M.R., B.M.P., R.G., H.Z., M.Z. and E.T. J.S., N.A., J.L.B., M.R., B.M.P. and H.Z. wrote the first draft of the paper and all authors contributed to subsequent revisions and approved the final version.

## Conflict of interest

None declared.

## Supplementary Material

dyab081_Supplementary_Data
