# Supplementary material for: Interactions between SARS-CoV-2 and influenza, and the impact of coinfection on disease severity: a test-negative design
Source: Int J Epidemiol. 2021 May 3;50(4):1124–33. doi: 10.1093/ije/dyab081 (PMC8135706; doi:10.1093/ije/dyab081)
Supplement: dyab081_Supplementary_Data [file dyab081_supplementary_data.zip › ije-2020-10-1980-File007.docx]

**Table S1.** Number and proportion of individuals testing for SARS-CoV-2 and influenza by demographic characteristics.

| **Characteristic** | | **Negative for SARS-CoV-2 and Influenza N (%)** | **SARS-CoV-2 positive/ Influenza negative N (%)** | **Influenza positive/ SARS-CoV-2 negative N (%)** | **SARS-CoV-2 and Influenza coinfection N (%)** |
| --- | --- | --- | --- | --- | --- |
| **Age** | **0 - 4** | 967 (7.0) | 38 (0.9) | 72 (7.3) | 0 (0.0) |
|  | **5-9** | 277 (2.0) | 6 (0.1) | 27 (2.7) | 2 (3.4) |
|  | **10 -19** | 811 (5.9) | 33 (0.7) | 146 (14.7) | 3 (5.2) |
|  | **20 - 29** | 1354 (9.8) | 163 (3.7) | 110 (11.1) | 1 (1.7) |
|  | **30 -39** | 1495 (10.9) | 296 (6.7) | 139 (14) | 7 (12.1) |
|  | **40 -49** | 1514 (11.0) | 425 (9.6) | 96 (9.7) | 2 (3.4) |
|  | **50 -59** | 1832 (13.3) | 660 (14.9) | 104 (10.5) | 5 (8.6) |
|  | **60 -69** | 1676 (12.2) | 668 (15) | 100 (10.1) | 6 (10.3) |
|  | **70 -79** | 1716 (12.5) | 829 (18.7) | 80 (8.1) | 14 (24.1) |
|  | **80 +** | 2115 (15.4) | 1325 (29.8) | 118 (11.9) | 18 (31.0) |
|  | **Unknown** | 6 (0.0) | 0 (0.0) | 0 (0.0) | 0 (0.0) |
| **Sex** | **Male** | 7053 (51.2) | 1935 (43.6) | 487 (49.1) | 20 (34.5) |
|  | **Female** | 6350 (46.1) | 2441 (54.9) | 488 (49.2) | 38 (65.5) |
|  | **Unknown** | 360 (2.6) | 67 (1.5) | 17 (1.7) | 0 (0.0) |
| **Ethnicity** | **White** | 10982 (79.8) | 3172 (71.4) | 739 (74.5) | 45 (77.6) |
|  | **Multiple Ethnicity Backgrounds** | 196 (1.4) | 57 (1.3) | 26 (2.6) | 1 (1.7) |
|  | **Black / Black British** | 433 (3.1) | 312 (7.0) | 27 (2.7) | 5 (8.6) |
|  | **Asian/ Asian British** | 1042 (7.6) | 506 (11.4) | 86 (8.7) | 4 (6.9) |
|  | **Any other Ethnic Group** | 391 (2.8) | 216 (4.9) | 39 (3.9) | 1 (1.7) |
|  | **Unknown** | 719 (5.2) | 180 (4.1) | 75 (7.6) | 2 (3.4) |
| **Comorbidity** | **Zero** | 6339 (46.1) | 1590 (35.8) | 567 (57.2) | 17 (29.3) |
|  | **1 +** | 7424 (53.9) | 2853 (64.2) | 425 (42.8) | 41 (70.7) |
