# Supplementary material for: Interactions between SARS-CoV-2 and influenza, and the impact of coinfection on disease severity: a test-negative design
Source: Int J Epidemiol. 2021 May 3;50(4):1124–33. doi: 10.1093/ije/dyab081 (PMC8135706; doi:10.1093/ije/dyab081)
Supplement: dyab081_Supplementary_Data [file dyab081_supplementary_data.zip › ije-2020-10-1980-File008.docx]

Figure S1: Sequential flow diagram of data linkages

SGSS (Second Generation Surveillance System) and DataMart SARS-CoV-2 and influenza samples taken between 01/01/2020 and 02/06/2020

n= 19,256

n with coinfection =58

Number of cases linked to PHE COVID-19 Deaths Dataset and / or with Demographic Batch Service (DBS) death record.

n= 2,469

n with coinfection = 25

Number of cases linked to a hospital admission record in Secondary Uses Service (SUS)

n= 12,253

n with coinfection = 58

Number of cases admitted to the intensive care unit (ICU

N= 1,666

N with coinfection = 7

Number of cases on a ventilator

n= 890

n with coinfection = 5
